# Nuclear Chaperone ASF1 is Required for Gametogenesis in *Arabidopsis thaliana*

**DOI:** 10.1038/s41598-019-50450-3

**Published:** 2019-09-27

**Authors:** Yunsook Min, Jennifer M. Frost, Yeonhee Choi

**Affiliations:** 1 Department of Biological Sciences, Seoul National University, Seoul, 08826 Korea; 2 Department of Plant and Microbial Biology, University of California, Berkeley, CA 94720 USA

**Keywords:** Plant sciences, Fertilization

## Abstract

Sexual reproduction in flowering plants is distinct from that in animals since gametogenesis requires production of haploid spores, which divide and differentiate into specialised gametophyte structures. Anti-Silencing Function 1 (ASF1) is a histone H3/H4 chaperone involved in chromatin remodeling during cell division, which we have found plays a critical role in gametophyte development in *Arabidopsis thaliana*. Using mutant alleles for the two ASF1 homologs, *asf1a* and *asf1b*, we show that ASF1 is required for successful development of gametophytes and acquisition of fertilisation competency. On the female side, reproductive failure is caused by aberrant development of ovules, leading to gamete degeneration. On the male side, we show both *in vitro* and *in vivo* that *asf1* mutant pollen tube growth is stunted, limiting fertilisation to ovules nearest the stigma. Consistent with ASF1 importance in gametogenesis, we show that *ASF1A* and *ASF1B* are expressed throughout female and male gametogenesis. We show that the gametogenesis defects can be corrected by ASF1A and ASF1B transgenes, and that ASF1A and ASF1B act redundantly. Thus, in contrast to the role of ASF1 in sporophytic cell cycle progression, our data indicate that during reproduction, ASF1 is required for the precise nuclei differentiation necessary for gametophyte maturation and fertilisation.

## Introduction

In flowering plants, gametogenesis requires several post-meiosis mitotic divisions to form differentiated gametophyte structures on both the female and male side. The mature female gametophyte consists of seven distinct nuclei that are characterised by diverse chromatin structure, reflecting their different fates. Notably, the homodiploid central cell nucleus has a decondensed chromatin structure and exhibits decreased DNA methylation compared to the egg cell^1,2^. This chromatin structure is necessary for the central cell to initiate genomic imprinting and form the endosperm of the developing seed^3,4^. Male mature gametophytes, or pollen grains, contain similarly distinct nuclei, whereby highly condensed sperm DNA contrasts with less compact and demethylated vegetative cell chromatin^5–7^. The structure of vegetative cell chromatin, aside from a vital role in germline transposon regulation^7,8^ is thought to also be important for downstream activity of the vegetative cell, including germination and growth of the pollen tube^9^.

The nucleosome unit of chromatin contains a basic histone octamer composed of a central H3/H4 heterotetramer with two flanking H2A/H2B heterodimers, and is associated with 146 bp of acidic DNA. Chromatin structure is dynamic, and the efficient removal and reassembly of nucleosomes is facilitated by histone chaperone proteins^10^. Since H2A/H2B dimers are located peripherally, they are not loaded onto DNA until after the H3/H4 tetramer has been deposited. During chromatin disassembly, H3/H4 removal from the DNA therefore takes place after H2A/H2B dimers are removed^11,12^. During DNA replication or transcription, H2A/H2B dimers are picked up by the FAcilitates Chromatin Transaction (FACT) complex^13–15^, followed by recruitment of anti-silencing function1 (ASF1) to chaperone H3/H4 tetramers. During replication in heterochromatin, chromatin assembly factor-1 (CAF-1), another H3/H4 chaperone, is required to interact with heterochromain protein 1 (HP1) which is enriched in centromeric and pericentromeric regions^16,17^.

The molecular function and biochemical mechanisms employed by histone chaperones have been well studied in animals and yeast. For example, vertebrate Asf1 plays a crucial role in replication-coupled chromatin assembly, cell cycle progression, and cellular viability^18,19^, and deletion of *ASF1* from fission yeast, fruit flies and vertebrate cells causes lethality^18,20,21^. Conversely, mutants for histone chaperones are often viable in *Arabidopsis thaliana,* providing excellent tools for investigating their functions *in vivo*, especially their roles in plant development and growth. The CAF-1 and FACT histone chaperones have been extensively studied in *Arabidopsis*. Mutations in *Arabidopsis* CAF-1 subunits *FASCIATA 1* (*FAS1*) and *FASCIATA* 2 (*FAS2*) exhibit pleiotropic developmental abnormalities, which can be linked to a variety of molecular consequences of CAF-1 loss, including open chromatin, dispersion of pericentromeric DNA and a reduced heterochromatin content. This in turn results in a dramatic increase in homologous recombination, leading to progressive and transgenerational losses of 45S rDNA and telomeric sequences^22–34^. During gametogenesis, post-meiotic mitoses are disrupted in *fas* mutant male gametophytes, resulting in the formation of only one sperm cell^35^. Mutations in FACT display pleiotropic defects in vegetative growth^36^, and during reproduction, FACT is required in the female gametophyte for DEMETER (DME)-mediated genomic imprinting of the *FLOWERING WAGENINGEN* (*FWA)* and *MEDEA* (*MEA*) genes^37^ and demethylation of DME targets by assisting its access specifically to heterochromatin^38^.

ASF1 is less well studied; originally discovered in budding yeast, it is a small, evolutionarily conserved histone chaperone of the H3/H4 family, shown in other organisms to be involved in several fundamental cellular processes. ASF1 is involved in both Replication-Coupled and Replication-Independent histone deposition pathways. During gene transcription in yeast and *Arabidopsis*, ASF1 association with chromatin results promotes H3K56 acetylation, in turn facilitating nucleosome disassembly, allowing RNA PolII recruitment to gene promoters^10,39,40^. ASF1-mediated chromatin remodeling is also required for DNA replication and gene silencing, and its role in nucleosome assembly and disassembly is notable in centromeric regions, where ASF1 displays overlapping function with the CenH3 chaperone Sim3 in fission yeast^41,42^. ASF1 has also been shown to be important during DNA damage checkpoint and repair^43–47^. ASF1 binds to newly synthesised H3/H4 dimers^48^ but physically occludes the H3/H4 tetramerization interface^48–50^ in yeast and human cells, suggesting that chromatin assembly involves the formation of an H3/H4 heterotetramer from two H3/H4 dimers, in which ASF1 plays a crucial role.

Two *Asf1* homologs (*Asf1a* and *Asf1b*) are found in most eukaryotes, except for *Drosophila* which has a single *asf1* gene^51^. Likewise, two gene homologs encoding *ASF1* were identified in rice and *Arabidopsis*^52^. *Arabidopsis* ASF1A and ASF1B act redundantly and have a multitude of roles in vegetative growth^53^. Double mutants exhibit drastic growth inhibition, associated with a reduction in cell number and expansion^53^. Double mutants also show S-phase arrest, reduction of endoreduplication, replication fork stalling and trigger double-strand-break (DSB) repair checkpoints, similar to CAF-1^53^. ASF1 is also involved in UV-induced DNA damage repair, possibly through interactions with histone acetyltransferases HAM1 and HAM2, and with acetylated histone H3/H4^54^. *ASF1* mutants also display reduced reproductive success, whereby flowers had reduced pollen grains and seed set, however, the precise reproductive processes impacted by ASF1 are unknown^53^. In this study, we analysed the entire reproductive life cycle of *Arabidopsis asf1a* and *asf1b* single and double homozygous mutants, and demonstrate that ASF1 is required for successful male and female gametophyte production.

## Results

### Mutations in *asf1a asf1b* double mutants cause defects during female gametogenesis

**Figure 1:**
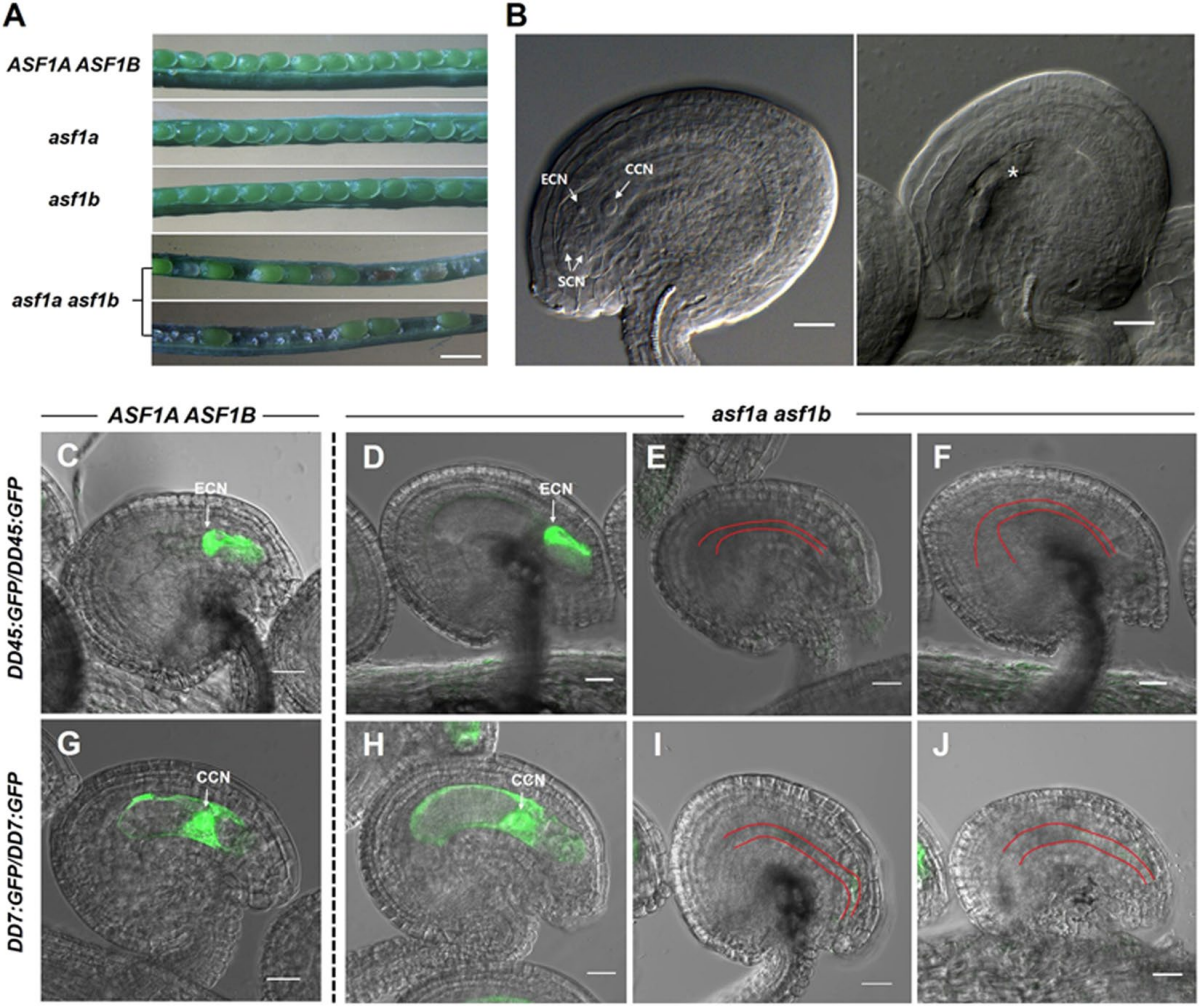
Characterisation of *asf1* mutants during reproduction. (**A**) Comparison of seed formation in wild-type and *asf1* mutant siliques. *asf1a asf1b* plants showing reduced seed-set and unfertilised ovules. (**B**) Normally developed (FG7, Left) ovule and defective ovule (Right) in *asf1a asf1b* plants containing a collapsed embryo sac before fertilisation. Asterisk indicates presumed female gamete cell nuclei. (**C–J**) *DD45:GFP* (egg cell marker) and *DD7:GFP* (central cell marker) transgenes were introduced into *asf1a asf1b* mutant plants. Images demonstrate the differences between non-arrested (**D**,**H**) and arrested (**E,F,I,J**) embryo sacs at the FG7 stage. (**D–F**) Ovules from the same pistil of *DD45:GFP/DD45:GFP; asf1a asf1b* plants. (**E,F**) DD45:GFP was not expressed in arrested mutant ovules. (**G–J**) Ovules from the same pistil of *DD7:GFP/DD7:GFP; asf1a asf1b* plants. (**I,J**) DD7:GFP was not expressed in arrested mutant ovules. (**C,G**) Ovules from wild-type siliques. DD45:GFP and DD7:GFP was expressed in the egg cell and central cell, respectively. CCN, central cell nucleus; ECN, egg cell nucleus; SCN, synergid cell nucleus. Red line indicates narrow and collapsed embryo sac. All images are created by merging a DIC image with a GFP image and photographed using confocal microscopy. Homozygous marker lines in the *asf1a asf1b* double mutant background were obtained in the segregating F2 generation. Representative microscopic images of the *DD45:GFP* or *DD7:GFP* transgene expression were reproducibly observed in >5 transgenic lines. Scale bars = 1 mm in (**A**) and 20 μm in (**B–J**).

The *Arabidopsis thaliana* genome encodes two ASF1 homologues, *ASF1A* (At1g66740) and *ASF1B* (At5g38110), which play roles in S-phase replication-dependent chromatin assembly^53^. We obtained T-DNA insertion mutants for *ASF1A* and *ASF1B* from the Arabidopsis Biological Resource Center (ABRC): *asf1a-2* (CS393977) and *asf1b-1* (Salk_105822C), both of which are loss-of-function alleles^53^. For the *asf1a-2* allele, we newly identified that the T-DNA insertion site is in intron 2, slightly downstream of the previously reported location in exon 2^53^ (Supplementary Fig. S1). Each mutant was crossed to produce double heterozygous plants and then self-pollinated. As previously described^53^, *asf1a* and *asf1b* single mutants did not show any distinct developmental defects under our growth conditions. However, *asf1a asf1b* double homozygous plants had low, but variable, fertility, exhibiting a range of viable seed numbers in any given silique (Fig. 1A, Table 1). Self-fertilised *asf1a asf1b* plants exhibited 73.9% seed or ovule abortion in total (n = 1,531) (Table 1), showing that ASF1 is required for successful reproduction.

**Table 1:** Analysis of *ASF1* mutants seed viability and complementation.

Parental Genotypes	NormalSeed(%)	Ovule Abortion(%)	Seed Abortion(%)	Total (n)
*ASF1A ASF1B*	93.6	5.1	1.3	968
*asf1a*	96.9	1.9	1.3	1497
*asf1b*	95.7	2.5	1.8	2503
*asf1a asf1b*	26.1	66.9	6.9	1531
*ProASF1A::ASF1A-GFP; asf1a asf1b*	97.1	2.5	0.5	442
*ProASF1B::ASF1B-GFP; asf1a asf1b*	98.1	1.1	0.7	1131

Abortion ratio = (No. of aborted seeds or ovules/No. of total seeds) × 100%.

To delineate the critical stage at which ASF acts during reproduction, we characterised cellular identities within *asf1a asf1b* double mutant ovules, introducing cell-specific markers (*DD45::GFP* for the egg cell and *DD7::GFP* for the central cell^55^) into double mutant plants using genetic crosses. We obtained homozygous marker lines in the *asf1a asf1b* double mutant background in the segregating F2 generation. *asf1a asf1b* ovules display variable morphology, where 2/3 are fully matured and appear normal, and 1/3 are narrow, collapsed and inviable. In the group of *asf1a asf1b* ovules that had fully matured (Fig. 1B left), we observed normal egg (Fig. 1D) and central cell fluorescence (Fig. 1H), however, none of the arrested ovules showed proper GFP expression for either marker within their collapsed embryo sacs (Fig. 1B right, E,F,I,J). We observed that 74% (n = 334) and 68% (n = 169) of the *asf1a asf1b* double mutant ovules displayed egg cell and central cell marker fluorescence, respectively, indicating that the majority of ovules with a fully matured appearance are viable (Supplementary Table. S1). However, the collapsed ovules have failed to undergo proper development or differentiation. Thus, approximately 30% of *asf1a asf1b* ovules have developmental defects before fertilization, and ASF1 is required for female gametogenesis, although the effect of the mutation is variable.

**Figure 2:**
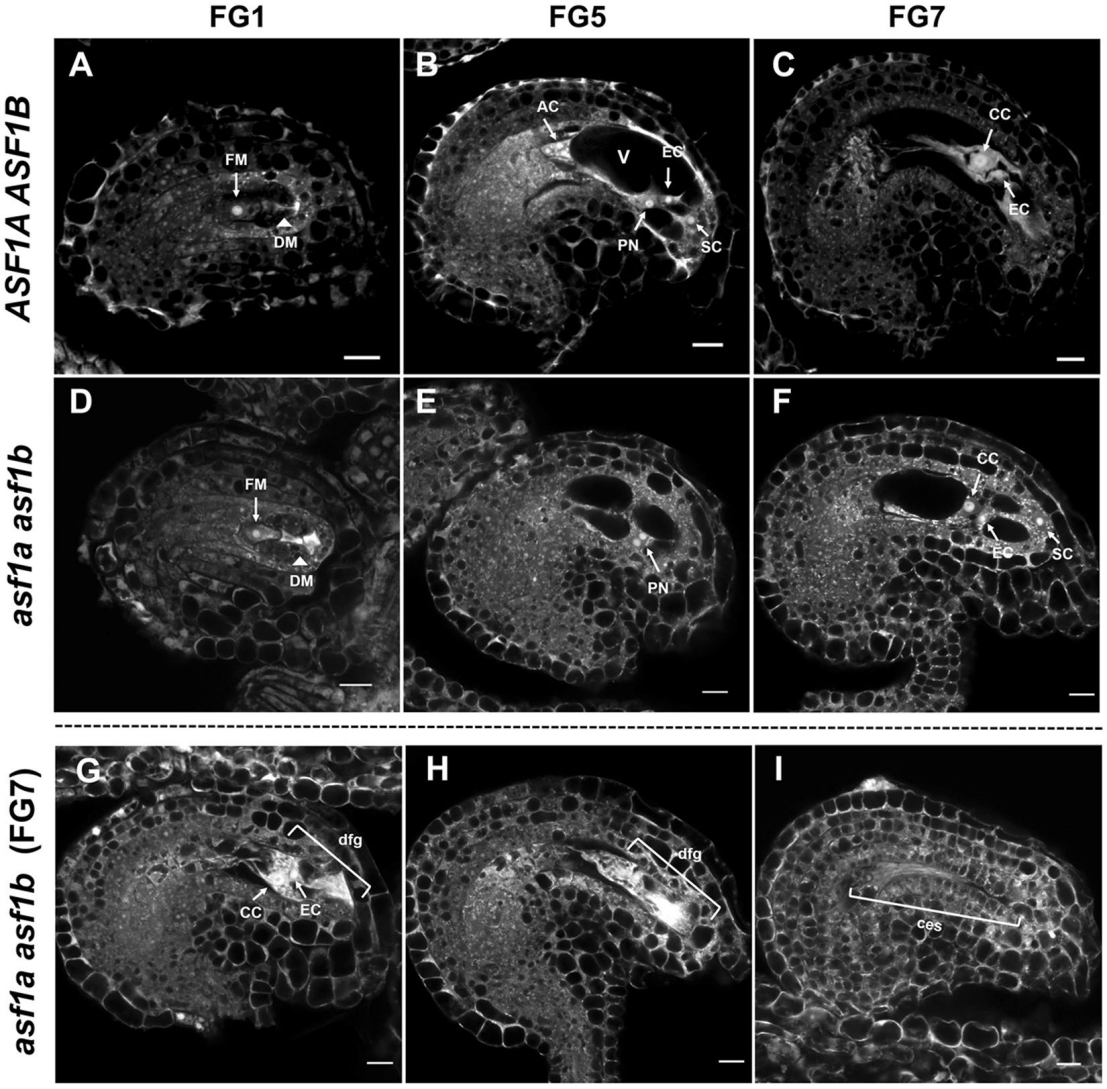
Defects in female gametogenesis of the *asf1a asf1b* mutants. (**A**) Wild-type ovules in the FG1 stage contained meiotic products, FM (arrow) and DM (arrowhead). (**B**) Three mitotic divisions from the FM occurred, and an eight-nucleated embryo sac containing a large central vacuole (V) was observed at the FG5 stage. (**C**) Central cell (CC) nucleus formed by fusion of two polar nuclei was observed. (**D–I**) *asf1a asf1b* mutants displayed normally developed ovules (**F**) and defective ovules (**G**–**I**) that showed degenerated and collapsed embryo sacs at the same FG7 period. Representative microscopic images of wild-type and *asf1a asf1b* ovules were reproducibly observed in >5 plants. DM, degenerated megaspore; FM, functional megaspore; EC, egg cell; PN, polar nucleus; AC, antipodal cell; CC, central cell; SC, synergid cell; V, vacuole; dgf, degenerated female gametophyte; ces, collapsed embryo sac. (**A**–**I**) Confocal microscopic images, in which the cytoplasm is displayed as gray, vacuoles as black, and nucleoli as white. Scale bars = 10 μm.

To ascertain the cause of ovule arrest in *asf1a asf1b* mutants, we analysed development of the female gametophyte prior to this stage using confocal microscopy. Megagametogenesis is characterised by mitotic divisions of the functional megaspore, formed during meiosis. At the first mitotic division, marking transition from FG1 to FG2, nuclei migrate to opposite poles, coincident with elongation of the embryo sac and formation of a large central vacuole. Two additional divisions form an eight-nuclear female gametophyte at FG5, followed by further nuclear migration and polar nuclei fusion to form the homo-diploid central cell at FG6. Two synergid cells and the egg are located at the micropylar pole, with three antipodal cells at the chalazal pole, which degenerate to form the mature female gametophyte at FG7 (Fig. 2A–C)^56^. *asf1a asf1b* ovules appeared to have comparable morphology to wild-type at FG1 (Fig. 2A,D) and displayed a similar length embryo sac to that of wild-type ovules during development (Fig. 2D–I). However, the width of the elongated embryo sacs in approximately 30% of the *asf1a asf1b* ovules was much narrower than wild-type and contained either some recognisable, but degenerated, FG7 nuclei (Fig. 2G,H), or no nuclei at all (Fig. 2I). These abnormal ovules did not develop further. The normal morphology of all FG1 *asf1a asf1b* ovules and presence of nuclei, albeit inviable ones that do not express appropriate cell lineage markers in the ~30% abnormal *asf1a asf1b* ovules, indicated to us that megasporogenesis proceeds normally and that mitotic divisions are occurring, but that the subsequent maturation and acquisition of cell identity are defective, resulting in a collapsed embryo sac without viable nuclei inside. Thus, ASF1 is required for megagametogenesis during female gametophyte development.

### Mutations in *ASF1* cause pollen defects during pollen germination

**Figure 3:**
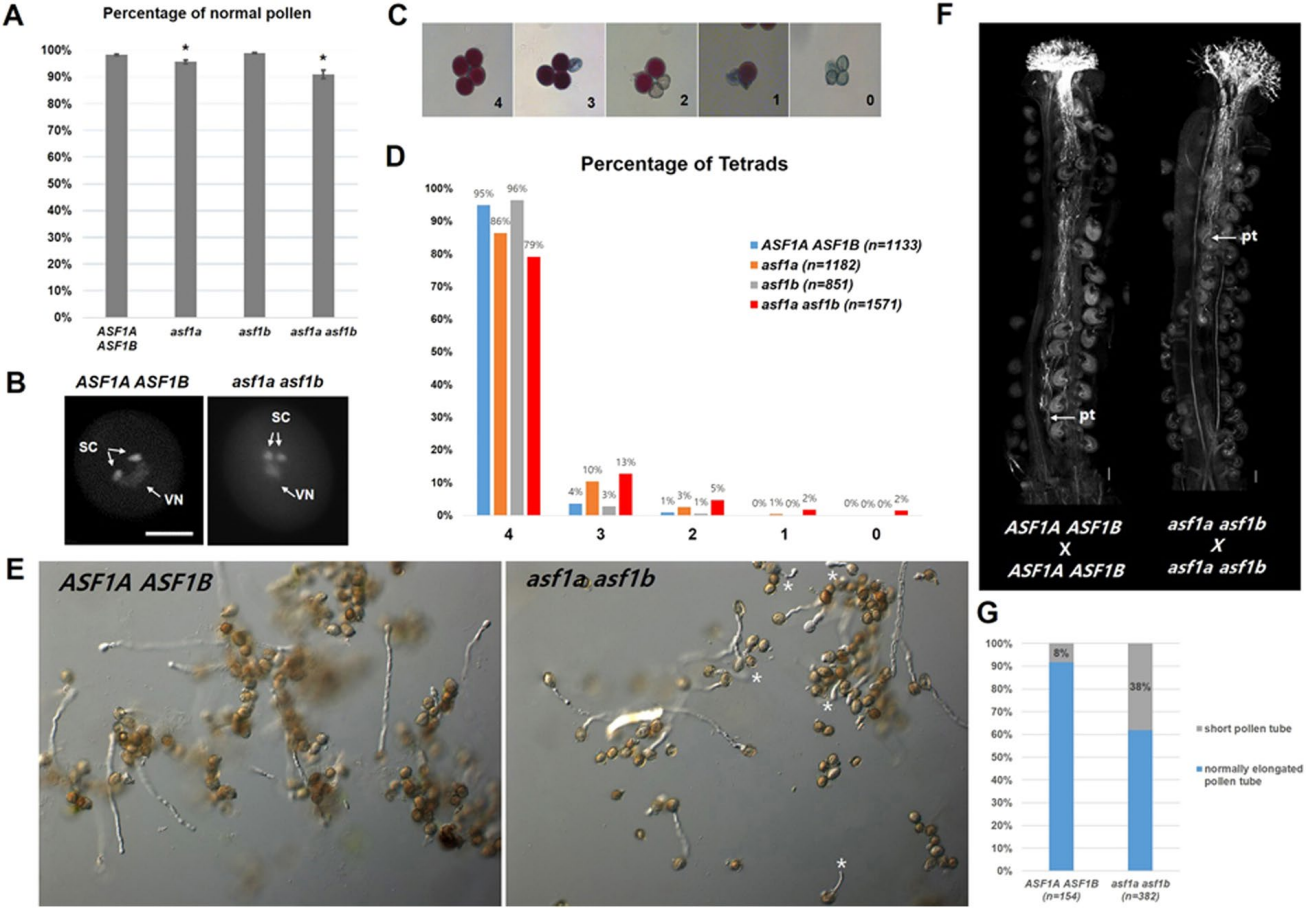
Analysis of microgametogenesis and pollen germination in *asf1a asf1b* mutant. (**A**) Percentage of viable pollen grains in wild-type versus asf1 mutants. Error bars represent mean ± SEM. Asterisks indicate the significance according to p-values from Student’s *t* tests between wild-type and *asf1* mutants: P < 0.01. (**B**) DAPI-stained pollen grain from wild-type (left) and *asf1a asf1b* (right) open flower. (**C**) DIC images of Alexander staining of the pollen in a qrt/qrt background. [4], a tetrad of four normal pollen grains; [3], a tetrad of three viable and one aborted pollen grains; [2], a tetrad of two viable and two aborted pollen grains; [1], a tetrad of one viable and three aborted pollen grains; [0], all aborted tetrad. (**D**) Percentage of tetrads containing 4, 3, 2, 1 or 0 normal pollen grains in wild-type versus *asf1* mutants. *asf1* mutant lines in the *quartet1-4* background were obtained in the segregating F2 generation of triple heterozygous plants. (**E**) DIC images of wild-type and *asf1a asf1b* mutant pollen tubes cultured at 22 °C *in vitro. asf1a asf1b* pollen showed short pollen tubes compared to the wild-type pollen tube. Asterisks indicate short pollen tubes of *asf1a asf1b* pollen. Images were taken with 10× magnification. (**F**) Aniline blue staining of pistils 24 hours after pollination (HAP). Growth pattern of the wild-type pollen tubes, showing that the pollen tubes almost had reached the end of the pistil. Growth pattern of the *asf1a asf1b* pollen tubes, showing that the pollen tubes only reached the middle of the pistil. The arrows indicate the pollen tubes. (**G**) Percentage of abnormal short pollen tube in wild type versus *asf1a asf1b* mutants. SC, sperm cell; VN, vegetative nucleus. pt, pollen tube. Scale bar = 10 µm in (**C**) and 100 µm in (**F**).

Self-fertilised *asf1a asf1b* plants exhibited much higher ovule abortion than is accounted for by our observations of aberrant female gametogenesis prior to fertilisation. Thus, we suspected that the remaining ovule abortion in developing *asf1a asf1b* siliques was due to a pollen defect, so that fertilisation does not occur normally. Indeed, a reduction in pollen grain number has been previously observed in *ASF1* mutant anthers^53^. We also observed a reduction in pollen grain number in *asf1a asf1b* anthers (Supplementary Fig. S2), however, when we compared selfed *asf1a asf1b* siliques with those from *asf1a asf1b* plants that we hand-pollinated (using a large excess of pollen grains) with *asf1a asf1b* pollen, ovule abortion incidence was not significantly different (Supplementary Fig. S3, p = 0.1454). This showed that a low pollen grain number was not contributing to the ovule abortion in *asf1a asf1b* mutants. To further investigate potential pollen defects, we measured pollen viability using Alexander’s staining, which can distinguish viable pollen (purple) from nonviable pollen (green). However, pollen viability of the *asf1a, asf1b* single mutants as well as *asf1a asf1b* double homozygous mutants was comparable to that of wild type (Figs 3A, S2).

We next asked whether microsporogenesis and microgametogenesis had proceeded as normal in *asf1a asf1b* male gametophytes. We performed a detailed analysis using DAPI (4′,6-diamidino-2-phenylindole) staining to examine whether pollen was tri-cellular, containing normal sperm and vegetative nuclei. In wild-type anthers, virtually all mature pollen was at the tri-cellular stage with fluorescently stained DNA contained within two punctate sperm nuclei and a diffuse vegetative nucleus. Consistent with the results from Alexander staining, most of the mature *asf1a asf1b* pollen grains also showed normal DAPI staining with all pollen at tri-cellular stage (Fig. 3B). Since pollen is produced in lower numbers, we hypothesise that ASF1 also impacts PMC formation, thereby leading to arrested PMC or subsequent abnormal PMI and PMII progression in *asf1a asf1b* mutants. However, our data show that microsporogenesis and microgametogenesis can be completed in *asf1a asf1b* mutants, producing viable tri-cellular pollen grains with two sperm nuclei and one vegetative nucleus.

To further investigate microgametogenesis in *asf1a asf1b* mutants, we used *QUARTET* (*qrt*) mutants, which fail to undergo microspore separation, releasing viable pollen tetrads (>95% shown by Alexander Staining (Fig. 3C)), and allowing us to examine the fate of each of the four progeny from individual pollen mother cells (PMCs)^57^. We generated *asf1a, asf1b*, and *asf1a asf1b* mutant plants in the *qrt1-4* homozygous mutant background by genetic crosses and then analyzed pollen generated from individual PMCs. Compared to wild type or *asf1b* plants, whose pollen grains were virtually all viable tetrads, *asf1a* single homozygous mutants (Fig. 3D, Orange bar, n = 1,182) and *asf1a asf1b* mutants (Fig. 3D, red bar, n = 1,571) exhibited a slight reduction for all viable tetrads. As in wild type and *asf1b* plants, 4:0 viable:nonviable ratios were the most common tetrads in *asf1a* and *asf1a asf1b* pollen (86% and 79%, respectively), but surprisingly, 3:1 viable:nonviable ratios of tetrads were also observed (10% and 13%, respectively). The slight reduction in 4:0 tetrad ratio in *asf1a* and *asf1a asf1b* pollen grains suggests that whilst the *asf1a* mutant phenotype is not completely penetrant, ASF1A has more dominant role compared to ASF1B during pollen formation. This could be, at least partially, explained by qRT-PCR result showing that *ASF1A* expression was increased approximately two times wild-type levels in *asf1b* mutants (Supplementary Fig. S4), thereby, possibly indicating that ASF1A upregulation may be compensating for the loss of ASF1B. However, in spite of a slight increase in 3:1 viable:nonviable ratios in *asf1a* and *asf1a asf1b* pollen, if we calculate the total number of individual viable pollen grains, we see a similar number of viable pollen grains between in *asf1a* and *asf1a asf1b* mutants (96% and 91%, respectively) compared to wild-type (Fig. 3A).

Since pollen development is only slightly affected by the *ASF1* mutation, we suspected the high percentage of ovule abortion in self-fertilised siliques could be due to defects in pollen germination. To examine this possibility, we tested pollen germination by spreading several drops of harvested pollen grains onto agar as described previously^58^. Wild type and *asf1a asf1b* pollen germinated at a similar rate. However, whilst wild-type pollen grains grew elongated pollen tubes in the germination conditions we used, many *asf1a asf1b* pollen grains did not fully elongate, and *asf1a asf1b* pollen tubes were thus frequently shorter than wild-type (* in Fig. 3E,G). To examine the overarching chromatin structure, and the dynamics of sperm and vegetative nuclei prior to fertilization, we used DAPI staining. Although we could not detect differences in chromatin condensation between wild type and mutant, we observed delayed migration of nuclei from the pollen grain into the pollen tube in *asf1a asf1b* mutants (Supplementary Fig. S5). By 2 h or even following overnight growth, the *asf1a asf1b* sperm cell nuclei remained in the pollen grains (Supplementary Fig. S5B,D) whereas migration of both the vegetative and sperm cell nuclei into the pollen tube was observed in wild-type pollen (Supplementary Fig. S5A,C). We also tracked *in vivo* pollen tube growth within pollinated pistils using aniline blue staining (Fig. 3F), finding that *asf1a asf1b* mutant pollen tubes were also shorter *in vivo*. Taken together, these data indicate that *asf1a asf1b* mutant pollen develops normally and is viable, but exhibits pollen tube elongation defects resulting in pollen tube stunting. As such, *ASF1* mutations seem to reduce reproductive success on the male side by reducing the rate of fertilisation.

### Reciprocal crosses show that ASF1 functions in both male and female gametophytes

Pollen tube growth is affected by signaling from the female gametophyte, in particular the synergid cells. In the absence of synergid cells, pollen tubes do not grow^59^. To exclude the possibility that the pollen tube phenotype was influenced by the loss of synergid cells in aberrant *asf1a asf1b* ovules, we observed siliques from reciprocal crosses between wild-type and *asf1a asf1b* mutants. When Col-0 wild-type plants were pollinated with *asf1a asf1b* mutant pollen, 31.4% ovules were aborted (n = 315) in the developing siliques, (Table 2, Supplementary Fig. S6). In addition, we noted that aborted ovules were more likely to reside at the end furthest from the stigma, consistent with a failure of short pollen tubes to reach this far (Supplementary Fig. S6, arrow). Therefore, around 1/3 of the reproductive defects in fertilisation in *asf1a asf1b* mutants are due to a male-failure of fertilisation caused by aberrant pollen tube growth. When *asf1a asf1b* mutants were used as the female, pollinated with wild-type pollen, we observed that 34.5% ovules aborted (n = 278) (Table 2, Supplementary Fig. S6). The ratio of ovule abortion:viable ovules observed in this genetic cross was comparable to the ratio of GFP-negative ovules from our analysis of cell-specific markers in *asf1a asf1b* mutant female gametophytes (Fig. 1C–J, Supplementary Table S1), indicating that the ovules without the appropriate FG7 nuclei are the ones that abort. The *asf1a asf1b* mutation thus impacts reproductive success to the same extent whether inherited form the male or the female side. When we hand-pollinated *asf1a asf1b* plants with *asf1a asf1b* mutant pollen, 59% of ovules were aborted before fertilisation (Table 2, Supplementary Fig. S6). This ratio reflects the sum of female and male defects seen in reciprocal crosses, showing that the negative effects of *asf1a asf1b* mutations downstream of gametophyte development are additive.

**Table 2:** Parental effects of *asf1a asf1b* alleles on seed development.

Parental Genotypes (female × male)	Normal Seed(%)	Aborted Seed(%)	Aborted Ovule(%)	Total (n)
*ASF1A ASF1B X ASF1A ASF1B*	94.5	1	4.5	201
*ASF1A ASF1B X asf1a asf1b*	57.8	10.8	31.4	315
*asf1a asf1b X ASF1A ASF1B*	55.4	10.1	34.5	278
*asf1a asf1b X asf1a asf1b*	22	18.9	59.1	264

F1 seeds of the reciprocal cross between *asf1a asf1b* and wild-type were counted to determine transmission efficiency of the mutant allele to the next generation through female and male gametes.

### ASF1 is required for cell cycle progression in the sporophytic phase of the *Arabidopsis* life cycle

**Figure 4:**
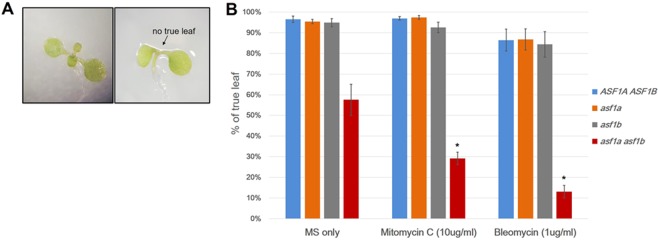
DNA damage sensitivity assays with *asf1* mutants. (**A**) Phenotypic morphology of 10-day-old seedlings with (left) and without (right, arrow) developing true leaves. (**B**) True leaf assay with seedlings treated with Mitomycin C or Bleomycin at the dosage indicated. The percentage of 10-day-old treated plants with true leaves was calculated in relation to MS only treated populations. Error bars indicate SEM of three biological replicates with 80–100 seedlings per each genotype. Asterisks indicate the significance between non-treated and drug treated *asf1a asf1b* according to p-values from Student’s *t* tests: P < 0.01.

In addition to the defects observed prior to fertilisation, after fertilisation 19% (n = 264) of developing seeds aborted during embryogenesis in hand-pollinated *asf1a asf1b* x *asf1a asf1b* F1 crosses. These aborting seeds showed delayed cell divisions in the embryo and endosperm when compared to the subset of *asf1a asf1b* normally developing seeds (Supplementary Fig. S7). ASF1 mutations in *Drosophila*, humans and yeast cause delayed cell division due to a defect in S-phase progression^19,47,48^. To measure the efficiency of S phase completion in *asf1a asf1b* plants, we used Bleomycin, which induces DSB in DNA, and Mitomycin C, which induces replication fork stalling^60,61^. We assessed the development of true leaves, which is a precise indicator of shoot-apical meristem cell divisions in post-germination seedlings. When grown on liquid MS medium containing 10ug/ml Mitomycin C, at least 89% wild-type and *asf1a* or *asf1b* mutant plants produced normal true leaves (Fig. 4A, left) at this concentration, but only 30% *asf1a asf1b* double mutant seedlings developed true leaves (Fig. 4A right, and 4B). When grown on Bleomycin (1ug/ml), *asf1* mutants were also more sensitive to this condition, with nearly 87% of *asf1a asf1b* double mutant plants displaying much reduced new leaf formation compared to with wild-type, *asf1a* or *asf1b* mutants. Finally, we measured the number of S-phase cells present in primary roots (grown in normal conditions) using 5-ethyl-2-deoxyuridine (EdU) staining. We observed that more cells are stained in *asf1a asf1b* mutant roots (Supplementary Fig. S8), as also reported previously^53^, indicating that the cells are stalled in S-phase, and thus the lack of ASF1 causes a delay in the completion of S-phase.

### Both *ASF1A* and *ASF1B* are expressed in female and male gametogenesis

**Figure 5:**
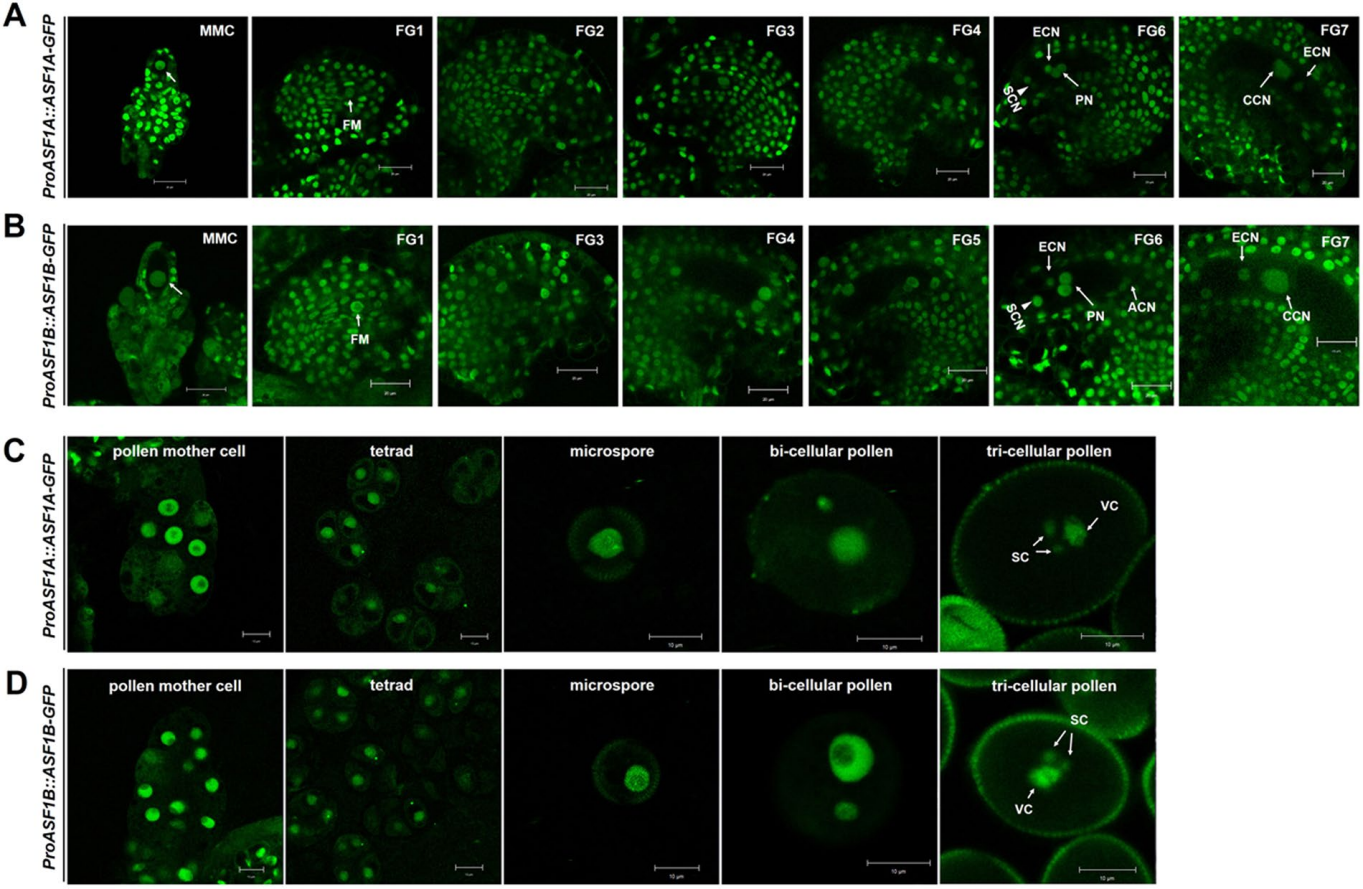
Expression pattern of ASF1A or ASF1B during female and male gametogenesis. (**A,C**) Representative microscopic images of *ProASF1A::ASF1A-GFP* transgene expression during reproductive stages. (**B,D**) Representative microscopic images of *ProASF1B::ASF1B-GFP* transgene expression during reproductive stages. *ASF1-GFP* transgenic lines in the wild-type background were observed in T2 generation. Representative microscopic images of *ASF1-GFP* transgene expression were reproducibly observed in more than 10 different transgenic lines. (**A,B**) Images of developing embryo sacs at MMC, and from FG1 to the FG7 mature stage. MMC, megaspore mother cell; FM, functional megaspore; SCN, synergid cell nucleus; ECN, egg cell nucleus; PN, polar nucleus; CCN, central cell nucleus; ACN, antipodal cell nucleus. (**C,D**) Images of developing pollen grains at PMC, tetrad, microspore, bi-cellular, and tri-cellular pollen. PMC, pollen mother cell, SC, sperm cell; VC, vegetative cell. All images are photographed using confocal microscopy. Scale bars = 20 µm (**A,B**), 10 µm (**C,D**).

To investigate the profile of *ASF1* expression during reproduction, and to assess whether the two homologs are expressed in a similar manner, we generated *ProASF1A::ASF1A-GFP* and *Pro ASF1B::ASF1B-GFP* transgenic plants containing *ASF1A* and *ASF1B* genomic sequences fused to GFP, respectively, under their own promoters. GFP fluorescence was observed during female gametophyte development using confocal microscopy. All nuclei at all developmental stages displayed ASF1-GFP expression. Both ASF1A-GFP and ASF1B-GFP were clearly detected in the diploid megaspore mother cell (MMC) before meiosis (Fig. 5A,B). GFP continued to be expressed in the functional megaspore after meiosis (FG1) and FG2 stage when the first mitotic division occurs during megagametogenesis^56^. GFP expression remained in mature female gametophytes (Fig. 5A,B). ASF1A and ASF1B expression were also observed, in surrounding maternal tissues, in agreement with the observations of Zhu *et al*.^53^, and consistent with a universal role for ASF1 as a histone chaperone in *Arabidopsis*.

On the male side, ASF1A:GFP and ASF1B:GFP were observed in the nucleus of the PMC and microspore, and continuously detected during PM I and PM II. At the tri-cellular stage, following PM II, ASF1A:GFP and ASF1B:GFP were present in both vegetative and generative cells (Fig. 5C,D). ASF1-GFP expression during gametogenesis, culminating in strong expression in the decondensed chromatin of the vegetative cell, is consistent with a role for ASF1 in the vegetative cell attaining pollen tube growth competency. Overall, these results support our genetic observations that ASF1 homologues are required during female and male gametogenesis.

### ASF1A and ASF1B are functionally redundant both in reproductive and vegetative growth

To ensure the *asf1a asf1b* mutant phenotypes observed during reproductive and vegetative growth were caused by mutations in *ASF1A* and *ASF1B* genes, and to test the redundancy of the two homologs, we introduced either *ProASF1A::ASF1A-GFP* or *ProASF1B::ASF1B-GFP* transgenes into *asf1a asf1b* double homozygous plants and analysed whether the defects were rescued. Our complementation analyses revealed that the transgenic plants produced either functional ASF1A or ASF1B, respectively, and were able to rescue the ovule abortion phenotype, as confirmed by counting ovules and seed sets in F2 plants of the crossed double mutants and transgenic plants (Supplementary Fig. S9A, Table 1). These transgenic plants in the *asf1a asf1b* mutant background produced functional ASF1A or ASF1B and showed comparable morphology to wild-type plants during vegetative growth (Supplementary Fig. S9B,C). These results show that the reproductive defects in *asf1a asf1b* plants are due to ASF1, and also provide further evidence that ASF1A and ASF1B are functionally and genetically redundant during reproductive and vegetative growth.

The phenotypes of *asf1a asf1b* are not completely penetrant, and in order to explore this, and also to test whether ASF1 mutation results in an  obvious defect in chromatin organisation, we measured whether chromatin regulatory proteins were dynamic in response to loss of ASF1 during reproduction. Comparing *asf1a asf1b* double mutant and wild-type floral buds with leaf tissue, we firstly measured the expression of histone chaperones CAF-1 (*fas1* and *fas2* alleles), HIRA and the H3/H4 histone chaperone Nuclear Autoantigenic Sperm Protein (NASP). We found that *FAS1* and *FAS*2 were significantly upregulated in mutant flowers compared to wild type, but not in leaves (Supplementary Fig. S10), indicating that these proteins were upregulated to compensate for the loss of ASF1, specifically in the gametophyte. In contrast, *HIRA* expression was upregulated in both *asf1a asf1b* double mutant floral buds and leaves, compared to wild-type (Supplementary Fig. S10), indicating that its expression is upregulated in response to ASF1 loss, but in a wider range of tissues. *NASP* expression did not change, but was higher in leaf tissue than in floral buds. We also measured expression of histone methyltransferase SET DOMAIN GROUP2 (SDG2), which deposits H3K4me3 in Arabidopsis, mediating vegetative cell DNA decondensation, and in turn pollen germination and pollen tube elongation^9^. We observed a significant decrease in expression of *SDG2* specifically in floral tissues. Finally, we assessed the levels of the FACT subunits; STRUCTURE SPECIFIC RECOGNITION PROTEIN 1 (SSRP1) and SUPPRESSOR of TY16 (SPT16). *SSRP1* levels appeared to increase in leaves, but not in floral buds, whereas *SPT16* exhibited a slight decrease in expression in *asf1a asf1b* double mutant floral buds, neither of which were significant (Supplementary Fig. 10).

In summary, in developing *asf1a asf1b* siliques, we observed abnormal ovules due to defects in ovule development (Supplementary Fig. S7D), normal but unfertilised ovules (Supplementary Fig. S7A) due to pollen tube formation and nuclei migration defects on the male side (Supplementary Fig. S7G), as well as delayed seeds that eventually abort (Supplementary Fig. S7B,E,H,I) due to decreased cell division in embryo and endosperm, which seems to be caused by a delay in S phase. We show that ASF1 homologs are both expressed throughout gametophytic development, and that the *asf1a asf1b* reproductive defects can be corrected by transgenic expression of ASF1A and B proteins. Finally, we show changes in expression of chromatin remodeling proteins that indicate a compensation to ameliorate the damaging effects of ASF1 loss in plant tissues. Taken together, our analyses reveal that ASF1A and ASF1B are required for proper reproductive development in *Arabidopsis* during both male and female gametophytic life stages, as well as during sporophytic development.

## Discussion

In this paper, we analysed the role of the ASF1 histone chaperone in reproduction in *Arabidopsis*. The protein products of both ASF1 homologs, ASF1A and ASF1B, were expressed throughout reproductive development and found to act redundantly, similarly to their activity in the sporophyte. In ASF1 double mutant plants (*asf1a* and *asf1b null*, or *asf1a asf1b)* there were a variety of gametophytic defects. In *asf1a asf1b* siliques, we observed aborted ovules with thin embryo sacs and degenerated nuclei (Figs 2G–I, S7D), ovules that had shriveled due to a lack of fertilisation (Supplementary Fig. S7A,G), as well as delayed seeds that eventually abort (Supplementary Fig. S7B,E,H,I). Following female megasporogenesis, *asf1a asf1b* ovules all achieved the FG1 stage without an obvious phenotype, indicating that this process had occurred normally, and that the defect manifests during post-meiotic mitoses. We observed a variety of aberrant ovule phenotypes, including some where some female gametophyte nuclei (i.e. synergid cells, egg cell, central cell and antipodal cells) had been formed and then degenerated, and did not express expected cell lineage markers (Figs 1 and 2). These ovules were also all characterised by a very thin embryo sac, and although we could not ascertain the dominant phenotype, it seems likely that the failure of maturation of the gametophyte cells leads to the collapse of the embryo sac.

ASF1 is required to rearrange chromatin during a variety of essential cellular processes, including DNA replication, transcription, gene silencing and DNA damage checkpoint and repair. We suggest ASF1 may be required to form the correct chromatin conformation during the FG1 to FG7 stages of nuclei division and differentiation during female gametogenesis. Cell fate is dictated by the chromatin landscape of cells, and in this way we suggest ASF1 could participate in rearrangement of chromatin to create the diverse cell identities of the *Arabidopsis* female gametophyte. Therefore, without ASF1, cell identity is lost (Fig. 1E,F,I,J) leading to nuclei degeneration and infertility. These data are consistent with observations of replication-independent activity of ASF1 in concert with HIRA in other organisms: reorganising chromatin to promote gene expression programs for muscle cell differentiation in mice^62^ and depositing histone H3.3 at promoters and enhancers of active and poised genes throughout the genome in HeLa cells^63^.

During male gametophyte development, the pollen produced was tri-cellular and viable, but exhibited a failure in pollen tube elongation, so that they were stunted, reducing the rate of fertilisation. This reduction in male fertility was also contributed to by a failure of migration of sperm and vegetative nuclei in the pollen tube. Thus, similar to the female gametophyte, though gametophyte nuclei were formed, they were unable to complete maturation, so that the vegetative cell did not produce a normal pollen tube, and they failed to migrate correctly. This is reminiscent of mutants of the SDG2 protein, which deposits H3K4me3 in *Arabidopsis*. Without SDG2, vegetative nucleus chromatin does not properly decondense, leading to compromised transcription activity, pollen germination and pollen tube elongation^9^. Indeed, *SDG2* expression was significantly decreased in *asf1a asf1b* floral tissues, perhaps in response to a reduction in appropriate chromatin formation. Pollen tube growth occurs by extension of the tip, and is regulated by calcium gradients and actin microfilaments. Elongation is also accompanied by rapid vacuole biogenesis, which are suggested to be important for this process^64^. We therefore found it intriguing that in the female gametophyte, the collapse of the embryo sac seemed to be caused by a failure in vacuole formation, although this may be secondary to the degeneration of gametophyte nuclei.

Previous work has analysed the role of ASF1 in the *Arabidopsis* sporophyte in detail^53^, and we add to this with our analysis of *asf1* mutant seeds. We find mutant seed development to be delayed and asynchronous, eventually resulting in seed abortion (Table 2, Supplementary Figs S6 and S7). ASF1 has both replication-dependent and -independent activities, however, since in the *Arabidopsis* sporophyte lifecycle, ASF1 is required for cell cycle progression, it is intriguing that we do not observe an overarching cell division phenotype during gametogenesis. It is possible that hidden defects in meiosis and the haploid mitoses of gametophyte development contribute to the phenotypes we observe downstream before fertilisation. Indeed, in CAF-1 (*fas*) *Arabidopsis* mutants, no meiotic phenotype was visible^65^. Similarly, normal meiosis was also observed when *AtASF1A* and *AtASF1B* were inactivated by RNAi^65^. However, Varas and colleagues go on to observe an increase in DSB during meiosis, shown by an increase in gH2AX foci^65^. The extra DSB undergo meiotic processing normally, being repaired by HR, hence the lack of a visible meiotic phenotype in the *fas1-4* single mutants^65^. We did not assess the frequency of DSB in our model, however, hidden haploid mitotic defects in chromatin organisation may have contributed to the gametophytic phenotypes we observe in *asf1* mutant gametogenesis. Similarly, cell cycle arrest in *fas* mutants only occurs during the haploid mitoses in male gametophyte development, which results in the formation of only one sperm cell^35^. Based on our genetic data, *asf1* mutant phenotypes manifest at a slightly later stage of gametophytic development than *fas1* mutant phenotypes, which is intriguing.

The phenotypic effects of ASF1 removal on both the male and female sides were not completely penetrant. Some *asf1a asf1b* mutant ovules developed normally through female and male gametogenesis, to produce normal seeds and seedlings. In sporophytic development too, *asf1a asf1b* selfed siliques contained approximately 1:1 normal to aborted seed ratios (Table 2, Supplementary Figs S3 and S6). Data from other organisms indicates that this may be due to redundancy between histone chaperone proteins. *Drosophila asf1* knock-down eggs exhibit only a partial requirement for ASF1, whereby the HIRA complex is still capable of nucleosome assembly in the male pronucleus, but this residual activity is not sufficient to ensure the timely assembly of paternal chromatin and pronuclear decondensation^51^. In *Xenopus* egg extracts, where other H3/H4 histone chaperones such as N1/N2, could possibly provide replicative histones to the CAF-1 complex^66^, ASF1 is dispensable for replication-dependent chromatin assembly. Therefore, we speculate that the absence of ASF1 is at least partially compensated in *Arabidopsis* by other histone chaperones such as CAF-1 and HIRA, consistent with our data showing increased expression of *FAS1, FAS2* and *HIRA* in *asf1a asf1b* double mutant floral buds (Supplementary Fig. S10).

In summary, we show that *Arabidopsis* ASF1 homologs are expressed throughout reproductive development, and are required for both male and female gametophyte maturation. We suggest this is due to its role in chromatin organisation during differentiation, and contrasts with the role of ASF1 in S-phase progression in the sporophyte.

## Methods

### Plant material and growth conditions

All *Arabidopsis thaliana* alleles were derived from the Columbia (Col-0) accession. *asf1a* and *asf1b* alleles correspond, respectively, to CS393977 (T3 generation) and Salk_105822C (T3 generation) T-DNA insertion strains from the Arabidopsis Biological Resource Center (ABRC) and the position of the T-DNA insertions was confirmed by PCR and sequencing. *asf1a asf1b* double mutants were obtained by crossing the two single mutants and subsequent self-pollination. No phenotypic variations were observed in subsequent several self-pollinated generations. All genotypes were determined by PCR analysis with the primers indicated in Supplementary Table S2. *Arabidopsis* plants were grown in a long-day (16 h light/8 h dark) photoperiod under cool white fluorescent light (100 μmole/m^2^/s) at 22 °C with 60% relative humidity.

### Seed-set analysis and whole-mount clearing

The number of normal seeds, aborted seeds, and undeveloped or unfertilised ovules was counted after DAP 8 to 10 siliques were dissected on a stereoscope. For whole mount clearing, pistils of mature FG7 stage and DAP 1 to 8 developing siliques were dissected and then, seeds and ovules were mounted in clearing solution (2.5 g chloral hydrate; 0.3 ml 100% glycerol; 0.7 ml distilled water). After incubation for several hours, samples were observed using an Axio Imager A1 microscope (Carl Zeiss) under DIC optics and were photographed using an AxioCam HRc camera (Carl Zeiss).

### Confocal laser scanning microscopic analysis (CLSM)

For the analysis of embryo sac development in wild type and *asf1a asf1b* mutants, CLSM analysis of ovules was performed as previously described^56^ with the following modifications; pistils of floral stage 6 to stage 12 were harvested. For fixation, dissected ovules were dipped in 4% glutaraldehyde (in 12.5 mM cacodylate, pH 6.9) under vacuum (~200 torr) for 20 min. The tissues were dehydrated through an ethanol series (10, 25, 50, 75, 90, 100, and 100% (v/v)) with 10 minutes per step. The dehydrated tissues were subsequently cleared in 2:1(v/v) benzyl benzoate:benzyl alcohol for 12 h, and then observed with a LSM700 (Carl Zeiss) confocal laser microscope.

### Pollen viability analysis using Alexander’s staining and DAPI staining

For analysis of pollen viability, pollen grains were mounted with Alexander’s staining solution (95% ethanol, 10 mL; Malachite green (1% in 95% ethanol), 1 mL; Fuchsin acid (1% in water), 5 mL; Orange G (1% in water), 0.5 mL; phenol, 5 g; chloral hydrate, 5 g; glacial acetic acid, 2 mL; glycerol, 25 mL; distilled water, 50 mL). To visualise nuclei in pollen grains, pollen was processed as described previously^67^. For DAPI staining, detached stamens were collected into a DAPI staining solution (0.1 M Tris-HCl, pH 7.0, 1 mM EDTA, 0.1% Triton X-100, and 0.4 ug/mL DAPI). The Alexander-stained slides and DAPI fluorescence were examined using an Axio Imager A1 microscope (Carl Zeiss) under DIC optics and were photographed using an AxioCam HRc camera (Carl Zeiss).

### Pollen Germination Assays *in vitro*

The pollen germination assay on solid medium from wild type and *asf1a asf1b* mutants was performed as previously described^58^ with a modification by spreading several drops of harvested pollen grains onto the surface of agar plates containing 1.5% agar (w/v). Images were observed using an Axio Imager A1 microscope (Carl Zeiss) under DIC optics and were photographed using an AxioCam HRc camera (Carl Zeiss) with 10x magnification.

### Aniline blue staining of pollen tubes

Pollen tubes were visualised using aniline blue staining as described previously^68^ with slight modifications. The pre-emasculated mature pistils were hand-pollinated with wild-type and *asf1a asf1b* pollen, respectively. The pollinated pistils were collected 24 hours after pollination (HAP) and fixed in fixative solution of 10% acetic acid in ethanol for 2 h at room temperature. The fixed pistils were hydrated by passing through an alcohol series (70, 50, 30 and 10% (v/v)) with 10 min per step and treated in softening solution of 1 M NaOH overnight. Then, the pistil tissues were washed in 50 mM KPO_4_ buffer and stained in aniline blue solution (0.1% aniline blue in 50 mM KPO_4_ buffer, pH 7.5) for 20 min. The stained pistils were observed and photographed with LSM700 (Carl Zeiss) confocal laser microscope under UV light.

### True leaf assay with mitomycin C and bleomycin

Seeds were plated in solid MS agar medium and stratified in the dark at 4 °C for 3 to 4 days and then transferred to the growth room. Using flame-sterilised and cooled forceps, 4-day-old seedlings were transferred to 6-well petri dishes containing 3 ml of liquid MS medium, either without (mock) or with a drug (10 μg/ml of mitomycin C or 1 μg/ml of bleomycin). After 5 days of incubation (with seedlings floating in liquid, but no shaking) in the illuminated growth room, the medium was removed from these 9-day-old seedlings, washed by flooding the plate with 5 ml of liquid media. Seedlings were transferred to solid MS plates using flame-sterilised and cooled forceps and allowed to recover for 24 h before analysis. Then, the percentage of plants with true leaves was scored. Sensitivity was indicated by the percentage of plants with true leaves.

### Vector construction of *ASF1:GFP* expression

A 1.5 kb fragment containing the full-length genomic DNA of *ASF1A* (1.2 kb) plus the 5′ upstream region (0.3 kb) and 2.6 kb fragment containing the full-length genomic DNA of *ASF1B* (1.6 kb) plus the 5′ upstream region (1 kb) were cloned into a *pBI-GFP* vector^3^. Gametophytes from plants expressing GFP fluorescence were analyzed using LSM700 (Carl Zeiss) CLSM.

### Quantitative RT-PCR

For expression analysis of *ASF1A* and *ASF1B* in wild type, *asf1a*, *asf1b*, and *asf1a asf1b* double mutants, inflorescence was collected. Total RNAs were extracted using liquid nitrogen and RNeasy Plant Mini Kit (QIAGEN). After gDNA wiped-out, cDNA was synthesized using the QuantiTect Reverse Transcription Kit (QIAGEN). qRT-PCR product was amplified using the iQ SYBR Green Supermix (Bio-Rad) on a CFX96 machine (Bio-Rad), and the data were analyzed using CFX Manager software (Bio-Rad). Relative transcript levels were normalized by the expression levels of *TUB*. For expression analysis of CAF-1*(FAS1, FAS2)*, *HIRA*, *SDG2*, FACT(*SPT16*, *SSRP1*) and *NASP* in wild type and *asf1a asf1b* double mutants, inflorescence and leaves were collected. RNA extraction, cDNA synthesis and qRT-PCR were performed as above. The primer sequences for qRT-PCR are listed in the Supplementary Table S2.

## Supplementary information

Supplementary Information
