# Supplementary material for: Nuclear Chaperone ASF1 is Required for Gametogenesis in Arabidopsis thaliana
Source: Sci Rep. 2019 Sep 27;9:13959. doi: 10.1038/s41598-019-50450-3 (PMC6764951; doi:10.1038/s41598-019-50450-3)
Supplement: Supplementary file 1 — Supplementary Information [file 41598_2019_50450_MOESM1_ESM.pdf]

Nuclear Chaperone ASF1 is Required for Gametogenesis in *Arabidopsis thaliana*

Yunsook Min[1], Jennifer M. Frost[2] & Yeonhee Choi [1]

[1]Department of Biological Sciences, Seoul National University, Seoul, 08826, Korea

[2]Department of Plant and Microbial Biology, University of California, Berkeley, CA 94720, USA

Correspondence and requests for materials should be addressed to Y.C. (email: yhc@snu.ac.kr)

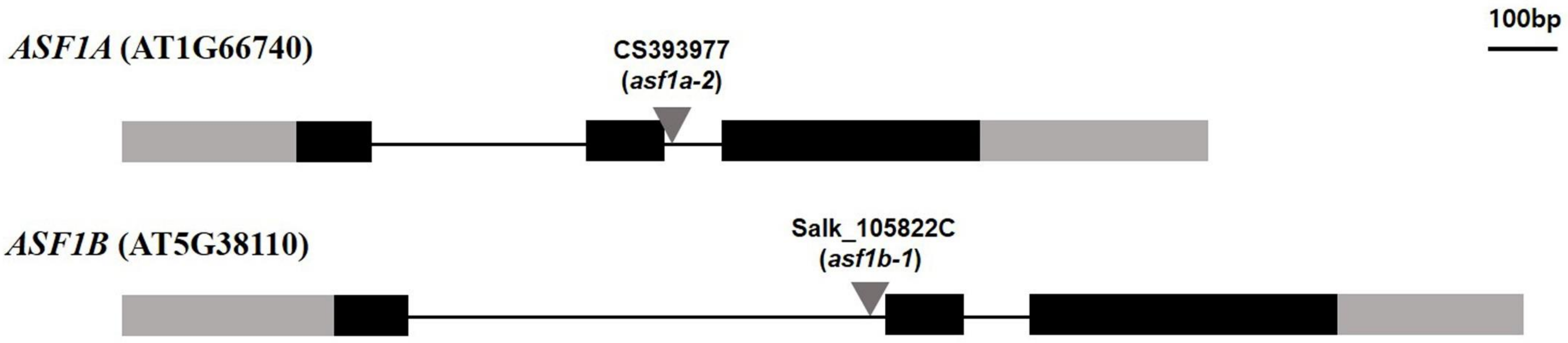

Supplementary Figure S1. Genomic structure of *ASF1A* and *ASF1B* and depicted T-DNA insertion.

Black box, translated exon; Gray box, untranslated exon; line, intron. The insertion sites of T-DNAs are marked by triangles.

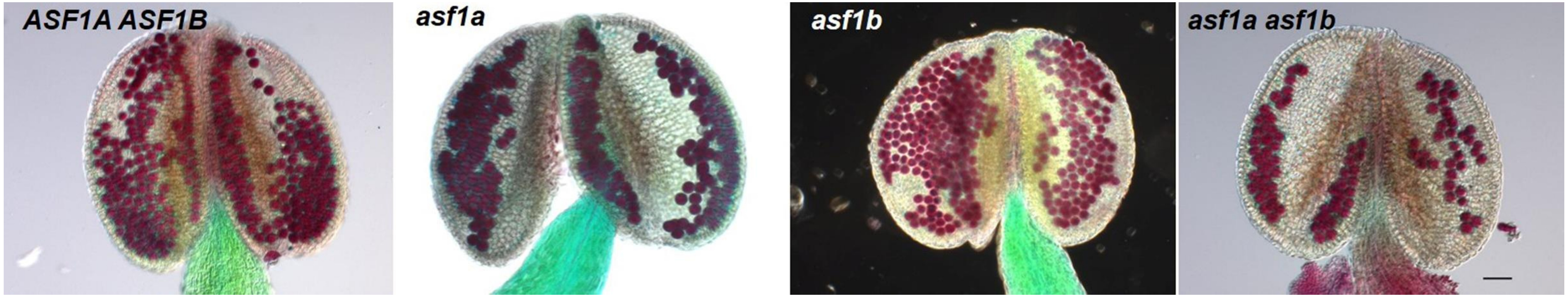

Supplementary Figure S2. Comparison of pollen viability of the wild-type and *asf1* mutant stamen.

Alexander staining of a wild-type and *asf1* mutant stamen. Purple stained pollen grains are viable. Scale bar = 50 µm

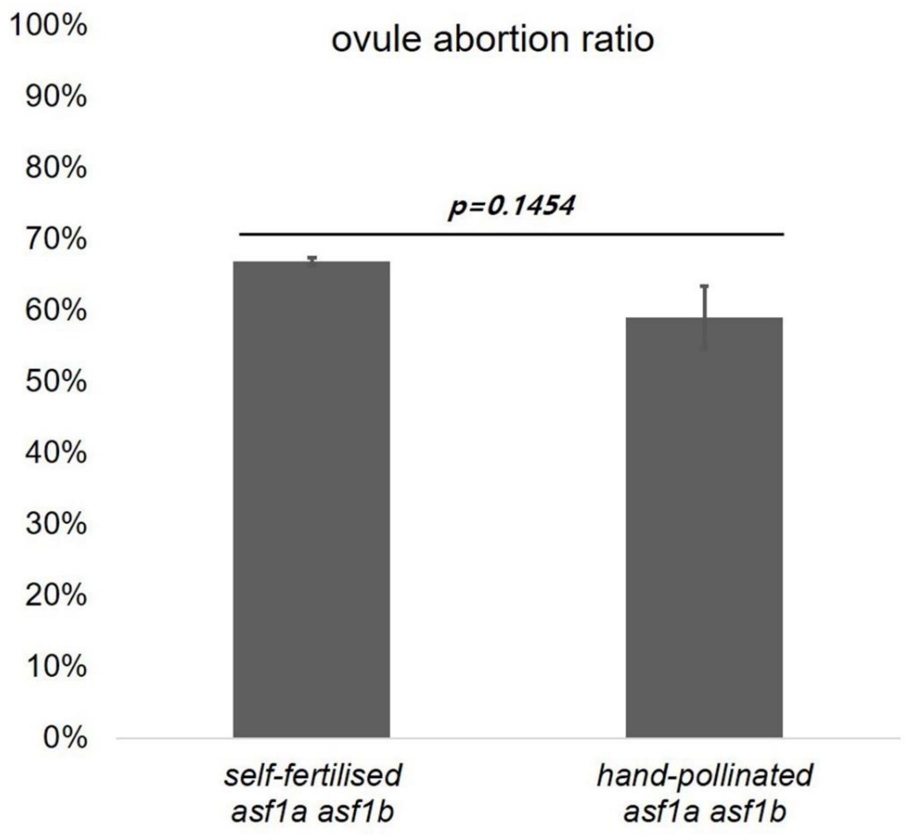

Supplementary Figure S3. Comparison of ovule abortion ratios between self-fertilised *asf1a asf1b* and hand-pollinated *asf1a asf1b*. This ovule abortion ratio has been reproduced from Table 1 and 2. Error bars represent mean ± SEM. p-value is for unpaired t-test with Welch's correction. A two-tailed t-test showed no significant difference of ovule abortion ratio between self-fertilised *asf1a asf1b* and hand-pollinated *asf1a asf1b*.

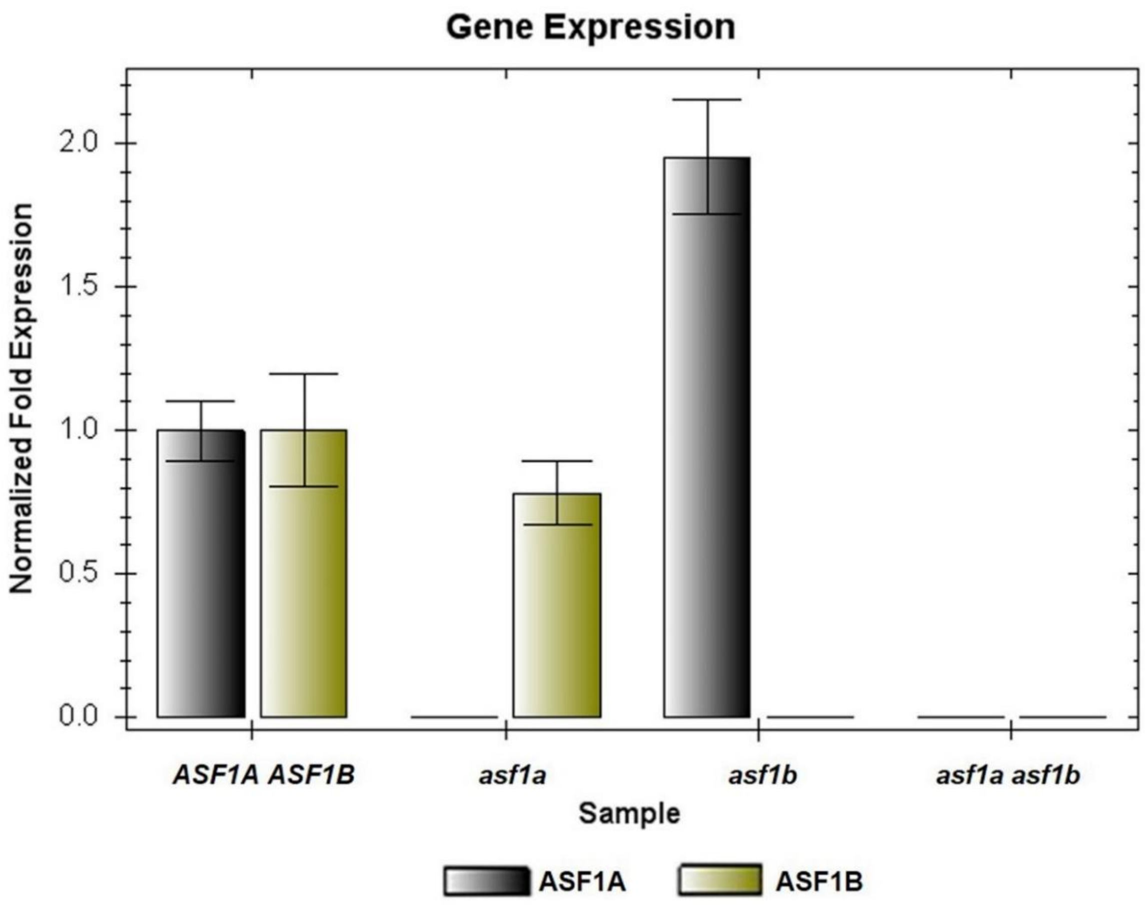

Supplementary Figure S4. Quantitative RT-PCR for *ASF1* and *ASF1B* in wild-type, *asf1a*, *asf1b* and *asf1a asf1b* mutants. qRT-PCR for *ASF1A* and *ASF1B* was performed with cDNAs from floral bud in wild-type, *asf1a*, *asf1b* and *asf1a asf1b* mutants, showing *ASF1A* expression increased in the *asf1b* mutants. Transcript levels were normalized to *TUB* transcript levels. Error bars indicate SEM of three biological replicates.

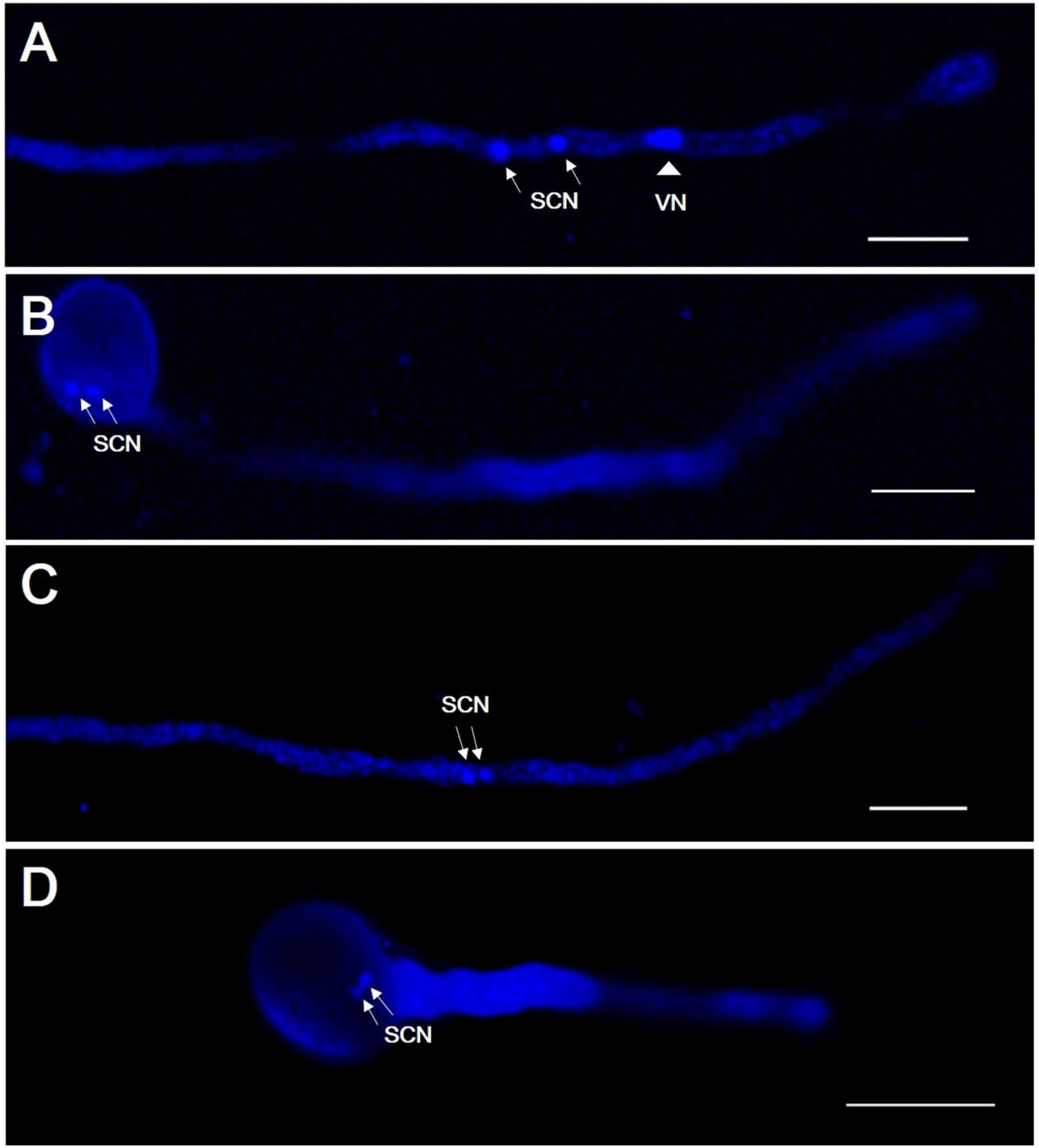

Supplementary Figure S5. DAPI staining of germinated pollen tube in wild type (A, C) and *asf1a asf1b* mutants (B, D). Migration of sperm and vegetative nuclei in wild-type pollen tube after overnight incubation (A) and 2h incubation (C) under pollen germination condition *in vitro*. Both sperm cell nuclei remained in the pollen grain of *asf1a asf1b* mutant even after overnight incubation (B) as well as 2h incubation (D). SCN, sperm cell nucleus; VN, vegetative nucleus. Scale bars = 20μm

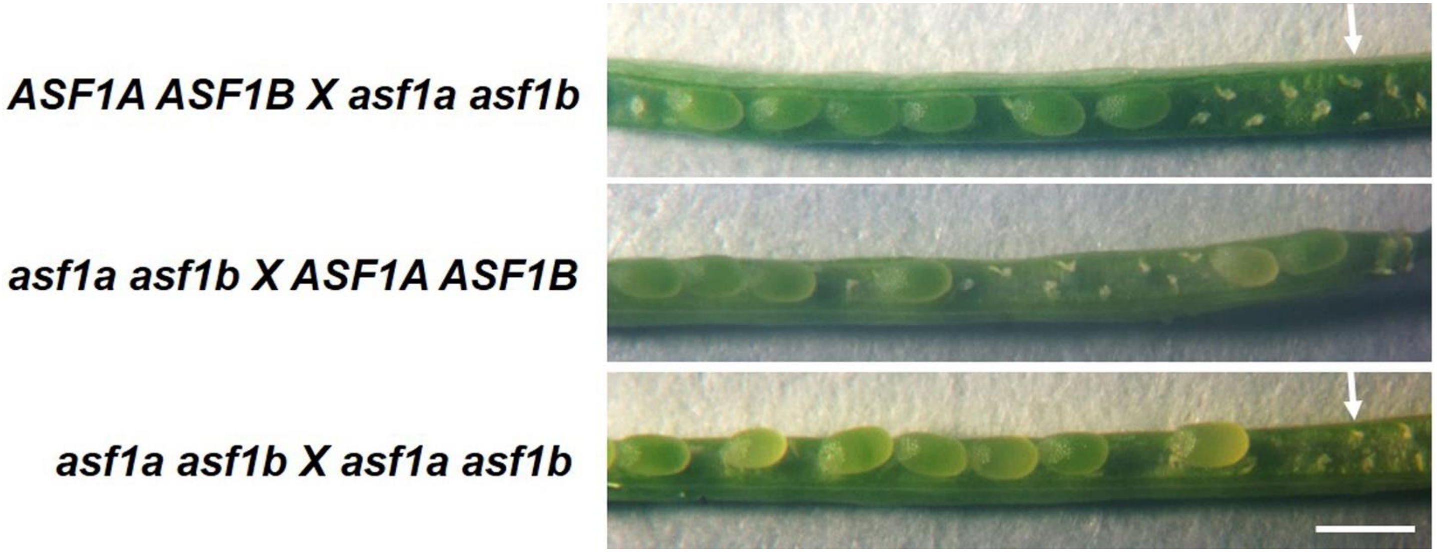

Supplementary Figure S6. Comparison of seed formation of the reciprocal crossed F1 siliques between wild-type and *asf1a asf1b*. Arrow indicates aborted ovules. Scale bar = 1mm

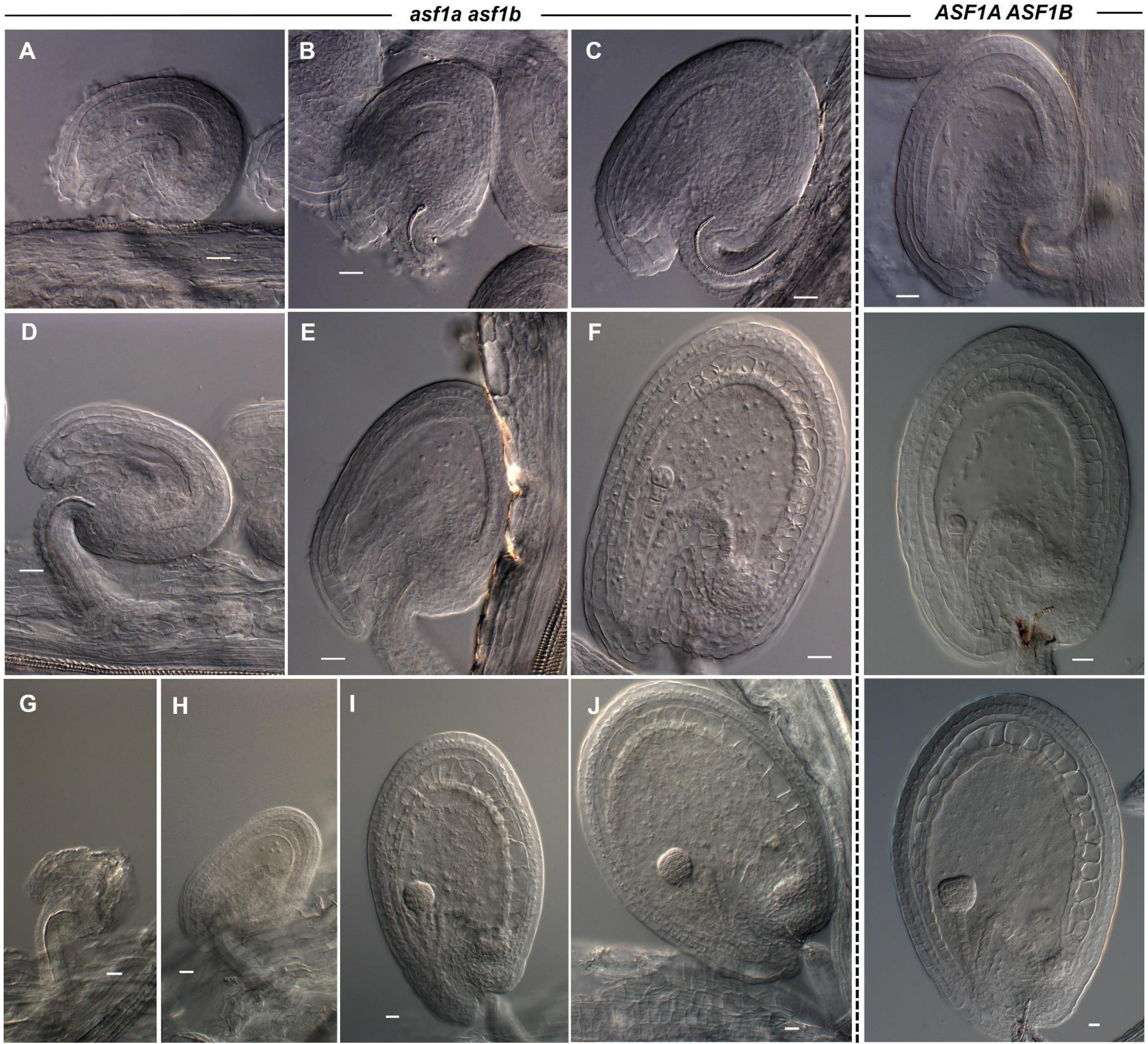

Supplementary Figure S7. Embryo development pattern of *asf1a asf1b* mutants after fertilization. **(A-C)** Endosperm development of *asf1a asf1b* plants after fertilization. *asf1a asf1b* plants containing unfertilized ovule (A) from the same pistil. **(D-F)** Representative seeds from the same pistil of *asf1a asf1b* plants. *asf1a asf1b* plants containing defective ovule (D) showed collapsed embryo sacs. **(G-J)** Delayed and defective seed development of *asf1a asf1b* plants from the same pistil. Embryo development of fertilized *asf1a asf1b* plants is delayed and asynchronous. Fertilized *asf1a asf1b* ovule showing the embryo (F, I, J) and endosperm nuclei (B,C,E,H). Scale bars = 20μm

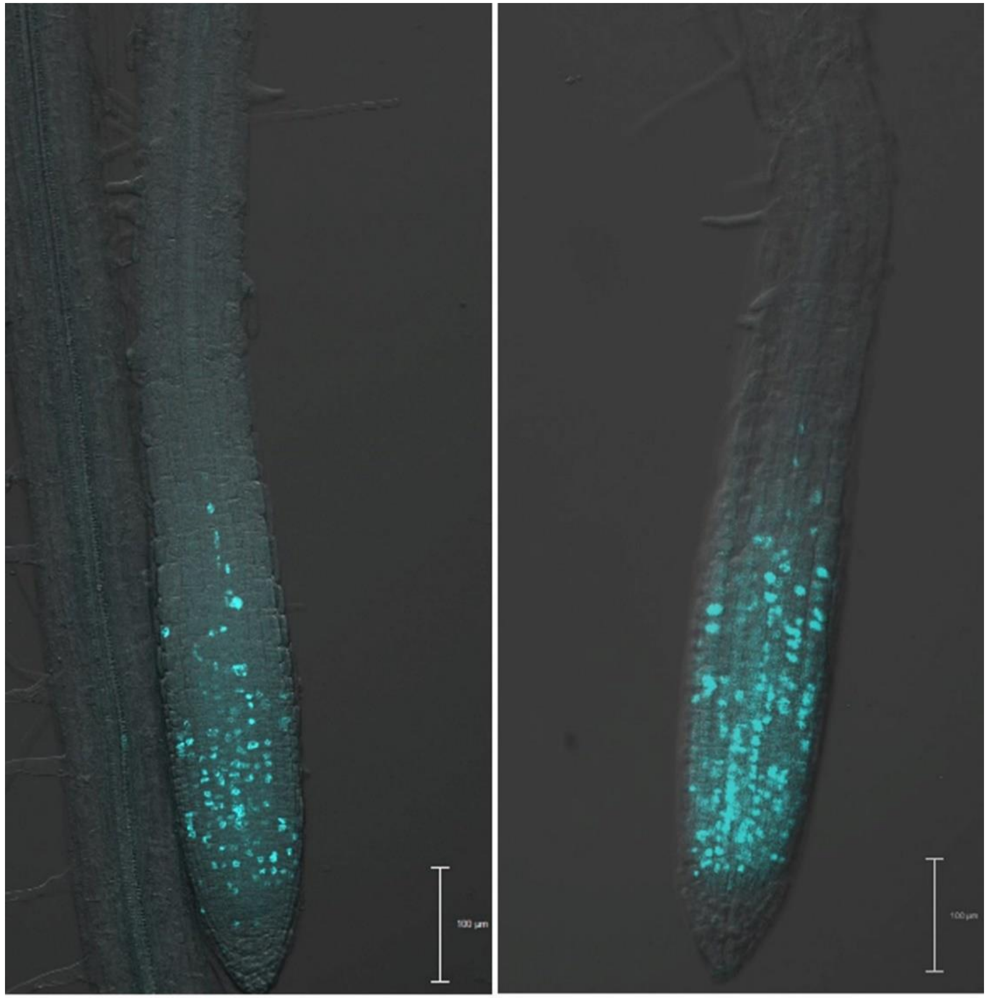

ASF1A ASF1B asf1a asf1b

Supplementary figure S8. *ASF1* is required for proper division of root cells. Comparison of EdU-staining on root tips of 8-day-old wild-type and *asf1a asf1b* plants. The EdU fluorescence images are created by merging with DIC image and photographed using confocal microscopy. Scale bars= 100µm

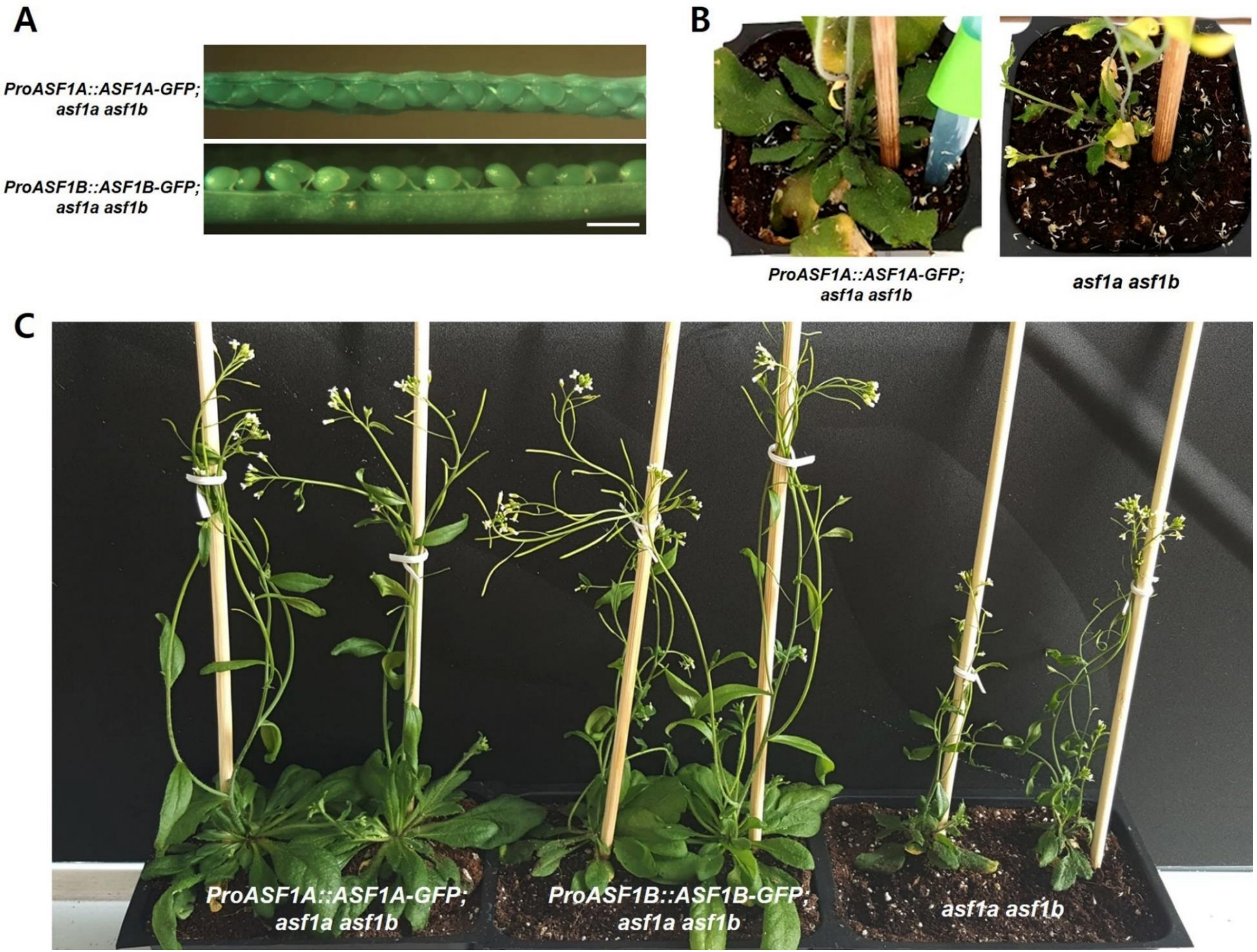

Supplementary Figure S9. Comparison of *asf1a asf1b* and the transgenic plants that expressing functional ASF1. **(A)** Dissected *asf1a asf1b* siliques that expressing functional ASF1A or ASF1B. **(B-C)** Vegetative phenotypes were rescued by *ProASF1A::ASF1A-GFP* or *ProASF1B::ASF1B-GFP*. Five different transgenic lines that expressing *ASF1-GFP* in the *asf1a asf1b* double mutant background were obtained in the segregating F2 generation and complementation was reproducibly observed in >5 plants per each line. Images were taken at 38 DAG (C).

Scale bar = 1mm in (A)

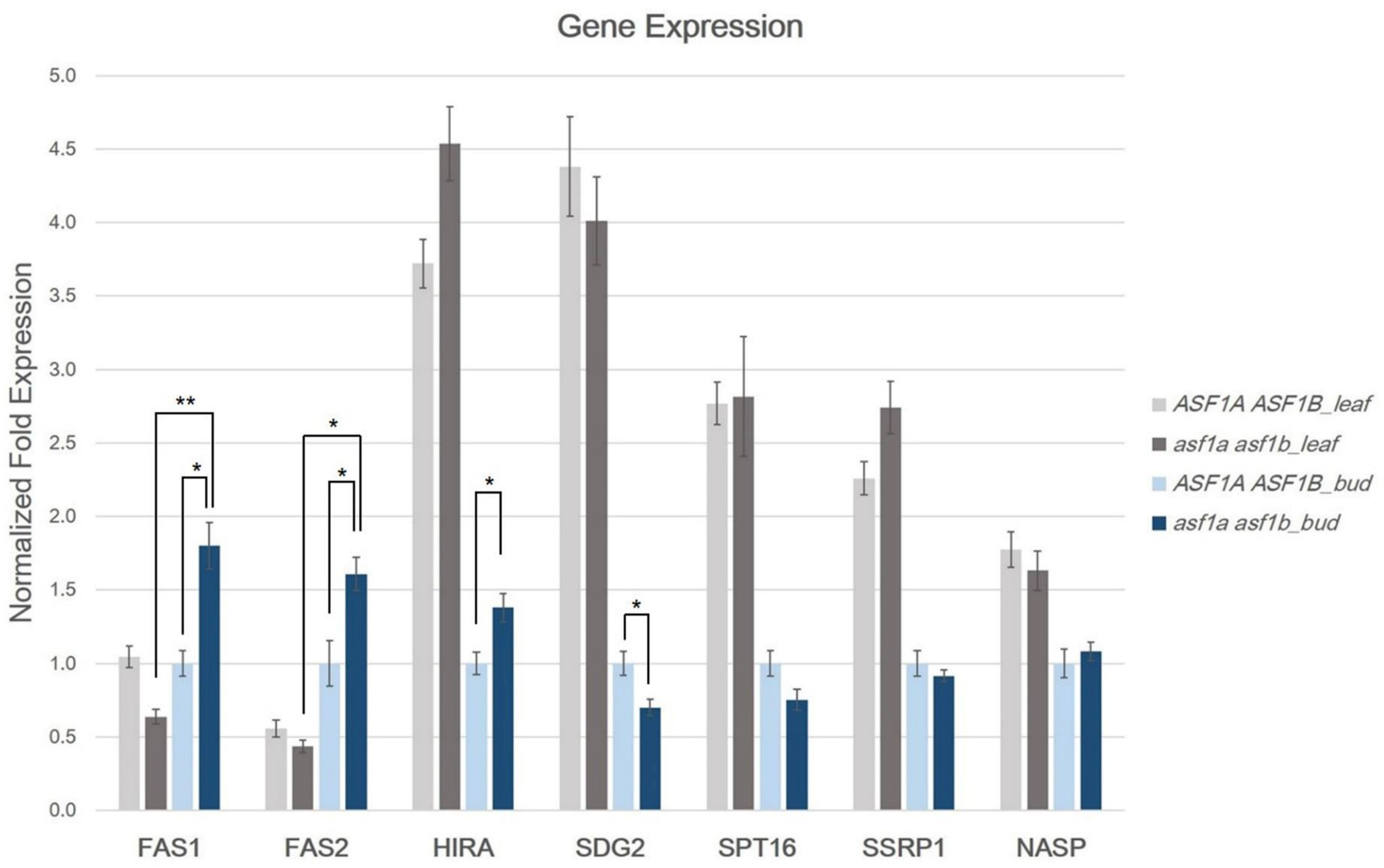

Supplementary Figure S10. Relative expression levels of chromatin related genes in wild type and *asf1a asf1b* mutants.

qRT-PCR for *FAS1*, *FAS2*, *HIRA*, *SDG2*, *SPT16*, *SSRP1* and *NASP* was performed with cDNAs from rosette leaf and floral bud in wild type and *asf1a asf1b* mutants. *FAS1*, *FAS2*, *and HIRA* showed increased expression whereas *SDG2* showed decreased expression in the *asf1a asf1b* floral tissue. Transcript levels were normalized to *TUB* transcript levels and the expression level of floral bud in wild type was used as control. Error bars indicate SEM of three biological replicates. Asterisks indicate the significance according to p-values from Student's *t* tests :* $P < 0.05$, ** $P < 0.005$.

Supplementary Table S1. Percentage of GFP expressing ovule in *asf1a asf1b* mutant at FG7 stage.

	**GFP positive ovule**	**Total (n)**
DD45:GFP/DD45:GFP; asf1a asf1b	248 (74%)	334
DD7:GFP/DD7:GFP; asf1a asf1b	115 (68%)	169

Supplementary Table S2. List of primer sequences used for T-DNA PCR, recombinant plasmid construction and qRT-PCR.

Gene analyzed	Primer name	Primer sequences
ASF1A (T-DNA PCR)	CS393977_F	GCATAATCCTGCTCCTTTTGTTAG
	CS393977_R	TTTGCCCAAAACATAAACATACAA
	LB_pAC161	ATATTGACCATCATACTCATTGC
ASF1B (T-DNA PCR)	Salk_105822C_F	GAGTTTTCCCAATTTAAATCGT
	Salk_105822C_R	GAAGCTTTTGTGGCTGCTCAAG
	LB_pROK2	GCGTGGACCGCTTGCTGCAACT
Pro0.3Kb:gASF1A:GFP	ASF1A_GFP_F	TGCCTGCAGGTCGACTTTGATGTCGTAAACACAAATG
	ASF1A_GFP_R	GCTCACCATGGATCCGCCGCCGCCTGATTCCTCAGGTTTTGGTTC
Pro1Kb:gASF1B:GFP	ASF1B_GFP_F	TGCCTGCAGGTCGACAAAAATAATTTTTGGCGCC
	ASF1B_GFP_R	GCTCACCATGGATCCGCCGCCGCCTGTCTCCTGGAGATTTTGTG
ASF1A (qRT-PCR)	ASF1A_qRT_F	TGTGCTTGTAGGGCCTGTTA
	ASF1A_qRT_R	ACCGATGATGTCTTCCTCCTG
ASF1B (qRT-PCR)	ASF1B_qRT_F	CCTGCTCCGTTTGTGAATCC
	ASF1B_qRT_R	TCCCAACGTTAACAGGACCA
FAS1	FAS1_qRT_F	TGGTAGCTGTGAAGAGTGCT
	FAS1_qRT_R	GAACGGAACTCGGCATGATT
FAS2	FAS2_qRT_F	CGATTACGATATCAAGCTATGG
	FAS2_qRT_R	TTCACTAGGATGCAACTTCC
HIRA	HIRA_qRT_F	ACTGCCGATGTGGTGGATCTTA
	HIRA_qRT_R	CCCAATCACTTGTTCGCCAT
SDG2	SDG2_qRT_F	CATGGGCCTGCAAGGTTATC
	SDG2_qRT_R	TCTCCAGCTCCTTGCTCAAT
SPT16	SPT16_qRT_F	AGCTGCCCTTTCTGTTGTTG
	SPT16_qRT_R	GCGGCTTTCTGACTCACATT
SSRP1	SSRP1_qRT_F	GTTATGCAGTTTGAAACAGACAC
	SSRP1_qRT_R	GCAACACGGTGGTGAATACTT
NASP	NASP_qRT_F	TCTCGAAATCAGGGTTGCAC
	NASP_qRT_R	CTTCTTGCCCACTTGAACTC
TUB	TUB_qRT_F	ATCGATTCCGTTCTCGATGT
	TUB_qRT_R	ATCCAGTTCCTCCTCCCAAC
